# Silicon supplementation affects mineral metabolism but not bone density or strength in male broilers

**DOI:** 10.1371/journal.pone.0243007

**Published:** 2020-12-07

**Authors:** Abby Pritchard, Cara Robison, Tristin Nguyen, Brian D. Nielsen

**Affiliations:** Department of Animal Science, Michigan State University, East Lansing, Michigan, United States of America; USDA-Agricultural Research Service, UNITED STATES

## Abstract

Because leg injuries produce welfare concerns and impact production for broilers, numerous interventions have been suggested as potential solutions. One mineral which may affect bone quality is silicon. The objective of this study was to determine if supplementing bioavailable silicon could affect bone morphology, mineralization, and strength without negatively influencing welfare and meat quality. Male broilers were raised from d 1 after hatching until 42 d of age and randomly assigned to treatment groups for silicon supplementation in water: Control (no supplement, C; n = 125), Normal (0.011 ml supplement/kg bodyweight, N; n = 125) and High (0.063 ml supplement/kg bodyweight, H; n = 125). Toe damage, footpad dermatitis, hock burn, and keel blisters were assessed on d 42. Blood samples were collected from wing veins for serum osteocalcin, pyridinoline cross-links, and mineral analysis. Clinical QCT scans and analysis were conducted immediately before four-point bending tests of tibias. Texture analysis was performed on cooked fillets. Silicon supplementation tended to increase daily water consumption in N and H as compared to C (*P* = 0.07). Footpad dermatitis and hock burn scores were higher in H than in N or C (*P* < 0.05 for both comparisons). Supplementation altered serum minerals (*P* < 0.001), but bone density, morphology, and strength measures were similar among groups. The highest level of supplementation in the current study on a kg bodyweight basis was above recommended intakes but below previous amounts demonstrating silicon’s positive influence on bone, indicating that previously suggested minimum thresholds need to be reevaluated. Factors such as growth rate and mechanical loading likely play a greater role in developing bone quality than trying to supplement on top of good basic nutrition alone.

## Introduction

Nearly 20 million metric tons of broiler meat per year are produced by the United States alone [1]. Due to genetic selection, commercial broiler chickens have rapid growth rates and high feed efficiency. As producers seek to increase production while reducing costs, birds will grow from about 40 g a day after hatching to around 3 kg in 42 days. This fast growth generates problems, ranging from pulmonary hypertension and sudden death syndrome [2] to leg deformities and fractures [3]. Because these birds have been selected for muscle gain and size, this selection creates a high muscle to bone ratio which puts additional strain on their legs. The fast growth also negatively impacts bone quality by increasing bone porosity [4] and decreasing ash and breaking strength per kg bodyweight [4, 5]. In addition to injuries and deformities, hock burns, keel blisters, and footpad dermatitis can cause additional pain, thereby reducing weight gain and efficiency, and may affect nearly 50% of birds in commercial operations [6, 7].

Because leg injuries produce welfare concerns and impact production [8, 9], numerous interventions have been suggested as potential solutions, many of which have aimed at improving bone mineral density (BMD). Because bone typically responds to loading [10], attempts at encouraging movement have been employed such as: adding perches [11], reducing stocking density [12], and increasing the photoperiod [13]. These methods have not convincingly improved leg health [14, 15] and have not been implemented in commercial systems. Frequently, changes to nutrition have been suggested, as feed is easier to manipulate than changes to the environment. Reducing metabolizable energy [16] or limiting overall feed intake [4] can improve bone quality by slowing growth rate, but producers do not want slowed growth. Varying concentrations and sources of calcium and other minerals [16–18] have positively influenced bone mineral content (BMC), BMD, and breaking strength. By manipulating these minerals, growth rate remains the same, but potential leg problems may be averted.

One mineral which may affect bone quality is silicon. Older studies showed that silicon deficiency in chicks and rats produces more porous, less mineralized bone [19–21]. Silicon intake in humans has been linked to greater BMD [22–24] and, in other species, silicon can increase bone formation markers such as osteocalcin [23] and reduce bone resorption markers [22], including pyridinoline [25]. In broilers, the inclusion of high levels of supplementary silicon has improved tibial ash content and breaking strength [26, 27]. Since silicon stimulates collagen synthesis, it may also impact meat quality, but feeding rice hulls, high in silicon, at levels up to 7.5 mg/kg feed did not increase thawing losses or shear force on thigh or breast meat in broilers [28]. Silicon may also reduce the instance of footpad, hock, and keel lesions as it has been associated with increased hydroxyproline content as a measure of collagen in calves’ skin [29]. To build upon these previous studies, the objective of this study was to determine if supplementing bioavailable silicon at two different concentrations could affect bone morphology, mineralization, and strength in a dose-dependent manner without negatively influencing welfare and meat quality. This study tested the hypothesis that increasing silicon concentrations would improve bone health measures as compared to controls and that these effects would be most prominent at the highest supplement concentration.

## Materials and methods

### Birds and management

All procedures were approved by the Michigan State University Animal Care and Use Committee (IACUC #: PROTO201800040). Male broilers (n = 375; Ross 708, Aviagen, Huntsville, AL) were raised from d 1 after hatching. Initially, broilers were divided into six 1.8 x 1.2 m pens of either 62 or 63 chicks per pen for brooding, but after the first week, chicks were divided randomly into 15 pens at 25 birds per pen. The photoperiod was stepped down from 24 h to 20 h over the course of the first 7 d on study and maintained at 20 h for the remainder of the study in accordance with the Ross Broiler Management Handbook [30]. Chicks had ad libitum access to water and a commercially-available starter-grower feed (Kent Nutrition Inc., Muscatine, IA) at all ages. Water was supplemented with a bioavailable silicon source based on randomly assigned treatment groups: Control (C; n = 125), Normal (N; n = 125) and High (H; n = 125). The C group received no supplementation. The dosage for the N treatment group represented the therapeutic dosage recommended by the manufacturer (Siliforce, Agro-Solutions, Nuth, Netherlands) for horses (0.011 ml supplement/kg bodyweight) while the H treatment group (0.063 ml supplement/kg bodyweight) received the dosage tested in a previous equine mineral balance study [31]. Water consumption with 15% wastage was calculated based upon age and projected weight according to estimates from previous broiler flocks [32], and silicon was added to the waters of the N and H treatment groups using an appropriately-sized syringe to ensure proper dosage.

### Pen weights and mortality

Birds were weighed twice weekly for dose adjustments. Five birds from each pen were randomly selected and weighed once weekly, and this weight was averaged per bird for pen dosing. Full pen weights were taken on a separate day once weekly to ensure accuracy. Mortality data included date, pen, and weight.

### Feed and water consumption

Feed was weighed in bag every time before being added to the storage containers (the empty bag weight was then subtracted). Containers were also weighed when empty and on a weekly basis to monitor consumption. At the start of the study, empty carboys with water lines and nipple drinkers were weighed and assigned to each pen. Water was measured in a graduated cylinder before being poured into the carboy. To measure consumption throughout, all water was drained out of the nipple drinkers and water lines back into the carboys, and carboys, lines, and drinkers were placed on scales and weights recorded. Water was then added while the carboys were still on the scales, and amount added was recorded every three days or as needed.

### Welfare scores

Toe damage, footpad dermatitis, hock burn, and keel blisters were assessed on d 42. Footpad dermatitis and hock burn were scored according to Bilgili et al. [33] where 0 indicated no lesions, 1 indicated mild irritation, and 2 indicated severe irritation. Toe damage included broken toes or nails was scored similarly to keel blisters as present (1) or not present (0).

### Blood samples

Blood samples were collected from wing veins with 22 G needles and syringes and placed into vacutainer serum separation tubes (BD Vacutainer^™^: SST^™^, Becton, Dickinson and Company, Franklin Lakes, NJ) on d 42 for serum osteocalcin (OC) and pyridinoline cross-links (PYD) analysis. Serum samples were centrifuged and placed into microcentrifuge tubes before freezing at -4°C. Serum samples were analyzed using OC (Microvue^™^ Osteocalcin EIA, Quidel, San Diego, CA) and PYD (Microvue^™^ Serum PYD EIA, Quidel, San Diego, CA) enzyme-linked immunosorbent assays. Silicon, boron, and calcium in plasma, as well as silicon concentrations in the supplement, were determined by inductively coupled plasma mass spectrometry (Agilent 7900 Inductively Coupled Plasma Mass Spectrometer, Agilent, Santa Clara, CA) by a certified laboratory (Michigan State University Veterinary Diagnostic Laboratory, Lansing, MI).

### Computed tomography scans

Clinical QCT scans and analysis were conducted according to Robison and Karcher [34] with the settings of 120 kV, 320 mAmp, and 0.625 mm slices. Briefly, right leg quarters were thawed in a chiller for 24 h prior to scanning. Legs were scanned with muscle and feathers intact and arranged in rows on plexiglass, and each scan included a solid calcium hydroxyapatite phantom (Image Analysis; Columbia, KY) of 0, 75, and 150 mg/cm^3^ calcium. A DICOM file of each row of leg quarters with an 11-cm field of view and bone algorithm was generated using Imageworks (General Electric Healthcare; Princeton, NJ) and imported for analysis into Mimics software (Materialise, Inc.; Plymouth, MI). The threshold for Hounsfield units (HU) was determined by applying a range from 200 to 600 HU with differences among thresholds of 25 HU based on thresholds used in the Robison and Karcher study [34]. Appropriate thresholds were set to 275 HU and 225 HU for the tibia and femur, respectively. After applying thresholds, whole bone volume and average HU were recorded for QCT BMC calculations. To determine bone density, average HU for each step of the calcium hydroxyapatite phantom was plotted against the known densities of the phantom to generate a standard curve. The following regression equation generated was used to calculate density in mg calcium hydroxyapatite/cm^3^: *y* = 0.7589*x* − 4.646, R^2^ = 0.99 where *y* is density in mg calcium hydroxyapatite/cm^3^ and *x* is HU. To calculate QCT BMC from CT scans, bone volume was multiplied by the density generated from the regression equation. Diameter, cross sectional area, and cortical area were measured.

### Bone breaking

After CT, four-point bending tests of tibias were conducted on a universal testing machine (Model 4202, Instron Corp., Canton, MA) according to ASABE Standards [35]. Tibias were placed with the palmar cortex under tension with a span between outer posts of 7 cm (outer span) and a span from inner post to outer post of 2 cm (inner span). A 10 kN load cell and a speed of 20 mm/min was used. One tibia was excluded from testing as it had been broken at CT. Fracture force, Young’s modulus, moment of inertia, and flexural rigidity of the tibias were determined. After breaking, caliper measurements were taken for bone length, fracture length, and distance of fracture from the proximal and distal end of the tibia. The proximal and distal locations of the fracture was defined as fracture start and fracture end, respectively. These points were used to calculate densities and other morphological data. Due to morphological changes within an individual tibia, moment of inertia and flexural rigidity were calculated at fracture start and fracture end.

Moment of inertia (I, mm^4^) was calculated using the following equation [35]:

$$I\;=\;0.049[(B\cdot D^{3})-(b\cdot d^{3})]$$

Where B is the outer lateromedial diameter, D is the outer dorsopalmar diameter, b is the lateromedial medullary diameter, and d is the dorsopalmar meduallary diameter. Young’s modulus of elasticity (E, N/mm^2^) was calculated as:

$$E\;=\;(\frac{F}{V})(\frac{a^{2}}{12I})(3L-4a)$$

Where $\left(\frac{F}{V}\right)$ is the slope of the force deformation curve from 1 to 2.5 mm extension, *a* is the inner span, and *L* is the outer span [36]. Flexural rigidity (EI, N∙mm^2^) was calculated as the product of the moment of inertia and Young’s modulus.

### Bone ash

Left leg quarters were autoclaved (733HCMC; Gentige, Wayne, NJ) at 121°C for 25 min in a method described by Cloft et al. (2018). After autoclaving, tissue and skin were removed, and tibias and femurs were separated (n = 50/bone/treatment). Each bone was cut into thirds, wrapped in cheesecloth, and placed into a modified soxhlet for ether-extraction for 12 to 24 h, after which they were dried at ambient temperature in a hood for 24 h and weighed. After ether extraction, bones were placed into crucibles and further dried in a DN-81 constant-temperature oven (American Scientific, Portland, OR) at 105°C for 24 h. Dry bone weights were obtained after this period, and crucibles containing bones were placed in an ash oven (Thermolyne 30400, Barnstead International, Dubuque, IA) overnight at 600°C. Ash was allowed to cool and weighed.

### Mineral analysis

Bone ash was ground and microwave digested (MARS5, CEM Corp., Matthews, NC) in duplicate with nitric acid (70% trace-metal grade; Fisher Scientific, Pittsburgh, PA) as described by Shaw et al. (2002). Twenty-four digests were randomly selected for analytical calcium, and calcium concentrations were determined by inductively coupled plasma mass spectrometry (7900 Inductively Coupled Plasma Mass Spectrometer, Agilent, Santa Clara, CA) by a certified laboratory (Michigan State University Veterinary Diagnostic Laboratory, Lansing, MI). Due to the large amount of calcium in bone, digests were diluted 100x to ensure readings were within range of the standard curve. The same inorganic standard was used for all samples, and digested peach leaves (Standard Reference Material^®^ 1547 Peach Leaves, National Institute of Standards and Technology, Gaithersburg, MD) served as controls. Total mineral amount within ash was determined by multiplying the concentration in mg/g by total amount of ash digested.

### Texture analysis

On d 42, whole keels were collected, tagged, and frozen at -20°C until analysis. For each day of analysis, keels were thawed for at least 24 h, and the right breast fillet (*Pectoralis major*) was cut from each keel and repacked in a plastic cooking bag. Fillets were cooked in batches, and a temperature probe was placed with a large left fillet to ensure that all fillets were cooked to an internal temperature of 71°C. Bags were weighed before and after cooking to determine cooking losses. After cooling, four 1-cm diameter cores were drilled from each fillet for shear force measurements. Each core was sheared perpendicular to muscle fiber direction by a Warner-Bratzler shear blade attachment at a test speed of 4 mm/sec with a 50 kg load cell on a TA-XT Plus Texture Analyzer (Texture Technologies Corp., Scarsdale, NY). Firmness and toughness were calculated from shear force using a macro within the texture analyzer software (Exponent Connect, Stable Micro Systems, Surrey, United Kingdom).

### Statistics

Residuals from continuous data were tested for normality and log-transformed as necessary for analysis. Pen weights, as well as water and feed consumption, were analyzed using a mixed model procedure (PROC MIXED) in SAS 9.4 (SAS Institute, Cary, NC) with fixed effects of treatment and date, repeated effect of date, and random effect of pen. Welfare scores were analyzed using the generalized linear mixed model procedure (PROC GLIMMIX) with the fixed effect of treatment and distribution set as either binary for toe damage and keel blisters or multinomial for footpad dermatitis and hock burn. The remainder of the measurements were analyzed using PROC MIXED with the fixed effect of treatment and random effect of pen. Significance was set at *P* < 0.05, and trends were considered at 0.05 ≤ *P* < 0.10. For significant effects, Tukey’s *post hoc* analysis was used for multiple comparisons.

## Results

### Pen weights, mortality, and feed and water consumption

**Fig 1:**
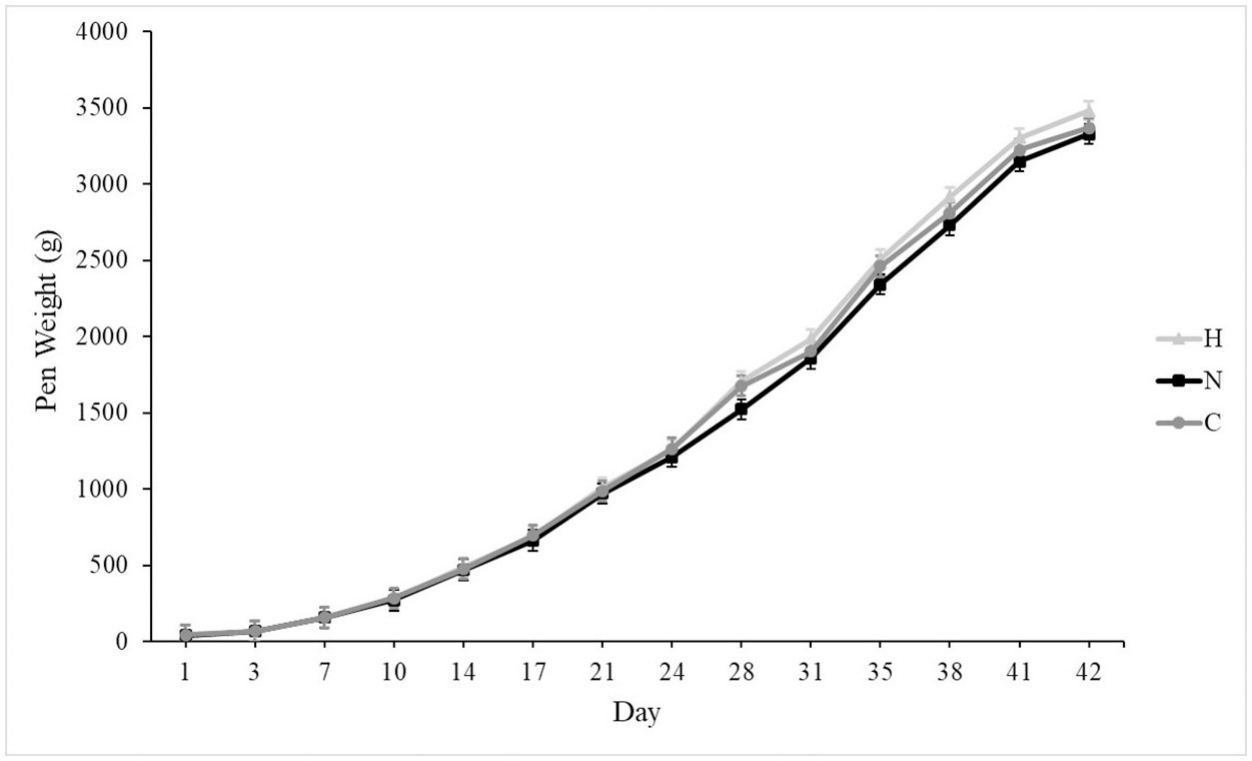
Mean bodyweight (± SE) of male broilers from d 1 after hatching to 42-d of age receiving from pens either no silicon supplementation (control, C, n = 5), normal supplementation (0.011 ml supplement/kg BW, normal, N, n = 5), or high supplementation (0.063 ml supplement/kg BW, High, H, n = 5). Weights were taken as pen weights and averaged over number of birds weighed.

**Fig 2:**
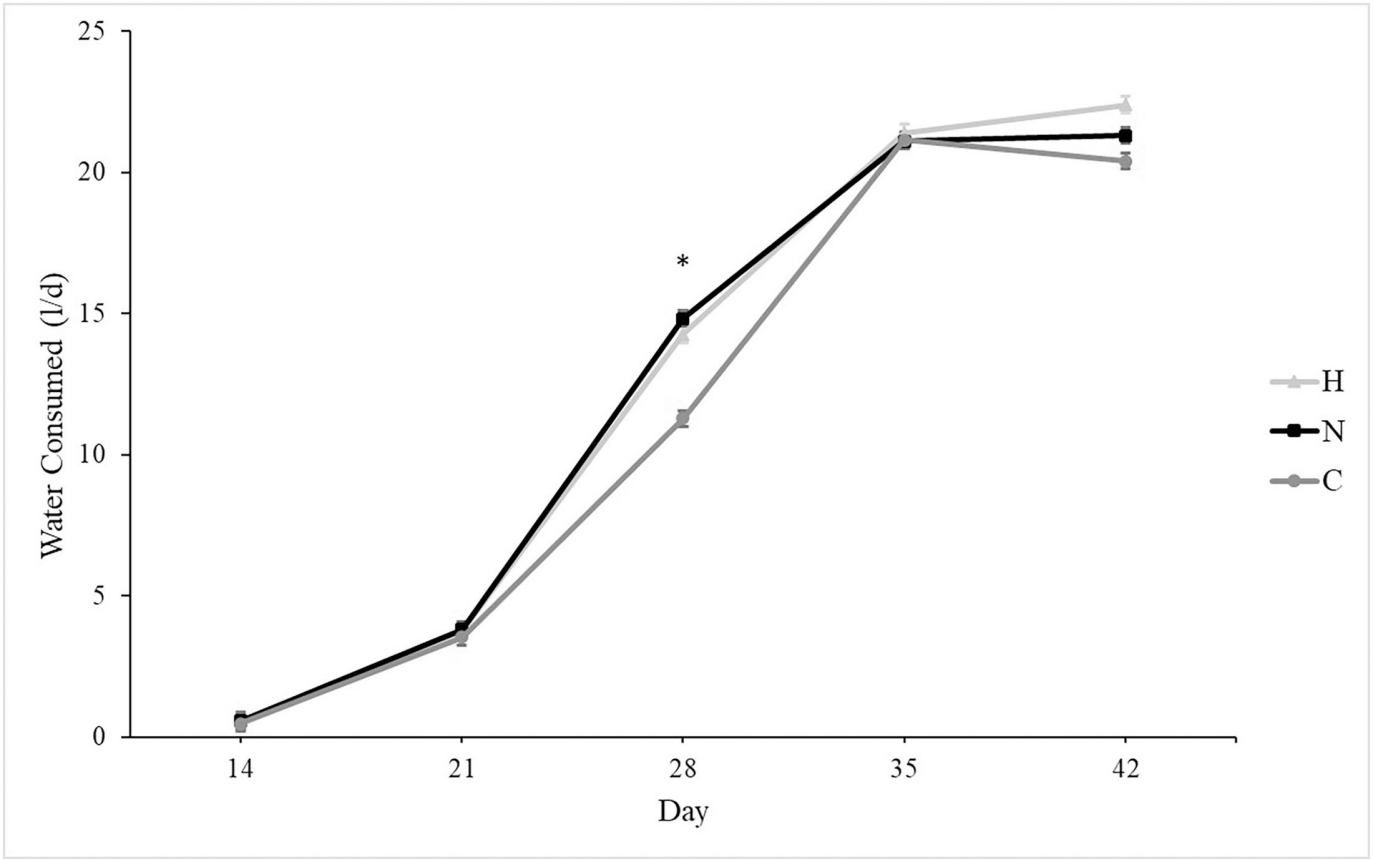
Mean water consumption (± SE) of broiler pens receiving either no silicon supplementation (control, C, n = 5), normal supplementation (0.011 ml supplement/kg BW, normal, N, n = 5), or high supplementation (0.063 ml supplement/kg BW, High, H, n = 5). Supplementation tended to increase water consumption (*P* = 0.07). *Indicates difference between treatment groups (N and H) and control (C, *P* < 0.005).

**Fig 3:**
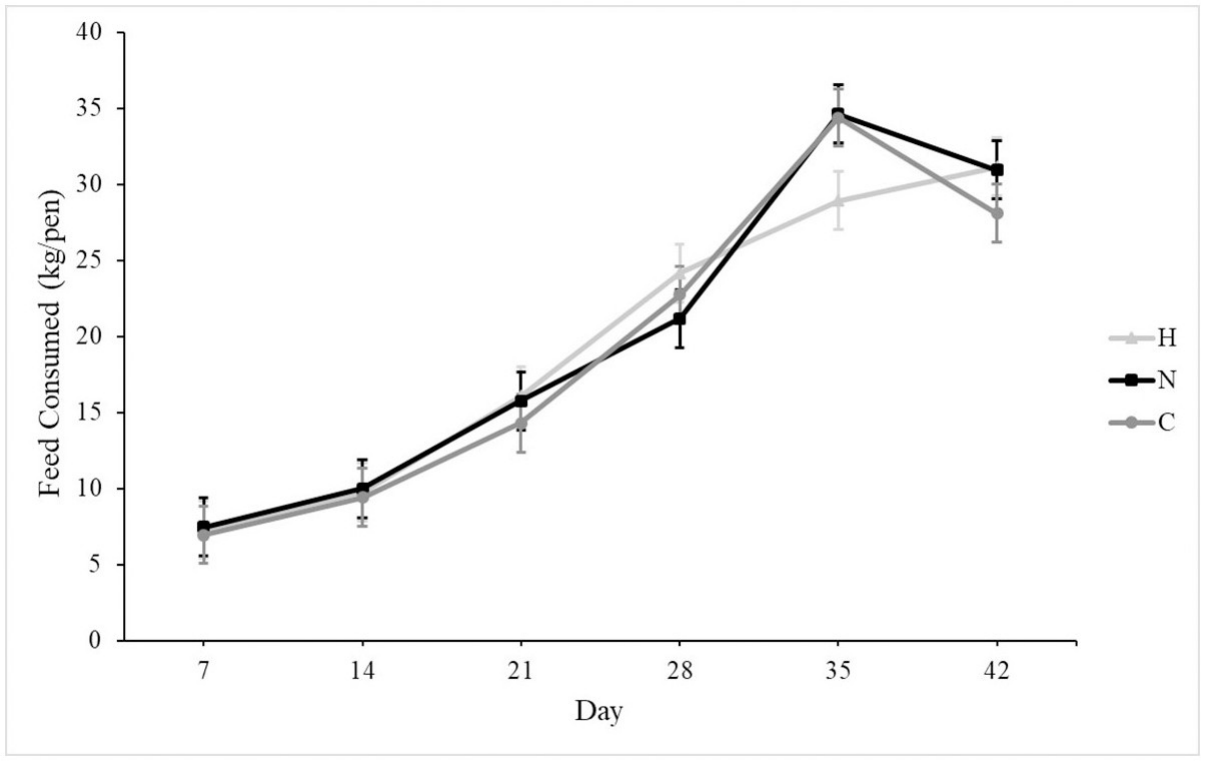
Mean weekly feed consumption (± SE) over six weeks of broiler pens receiving either no silicon supplementation (control, C, n = 5), normal supplementation (0.011 ml supplement/kg BW, normal, N, n = 5), or high supplementation (0.063 ml supplement/kg BW, High, H, n = 5).

At placement, all groups weighed similarly, and growth rates were similar among groups (Fig 1). On d 42, all groups weighed similarly. Mortality was unaffected by group, and mean mortality was 12.5 ± 0.02%. Silicon supplementation tended to increase daily water consumption in N and H as compared to C (Fig 2; *P* = 0.07). Overall feed consumption was similar among groups (Fig 3), and both feed and water consumption increased over time (*P* < 0.001 for both). Silicon intake from feed was 2.5 mg silicon/kg, and the supplement provided 8,988 mg silicon/l. Treatment did not affect feed conversion (C: 0.75 ± 0.03, N: 0.71 ± 0.03, H: 0.77 ± 0.03).

### Welfare scores

**Fig 4:**
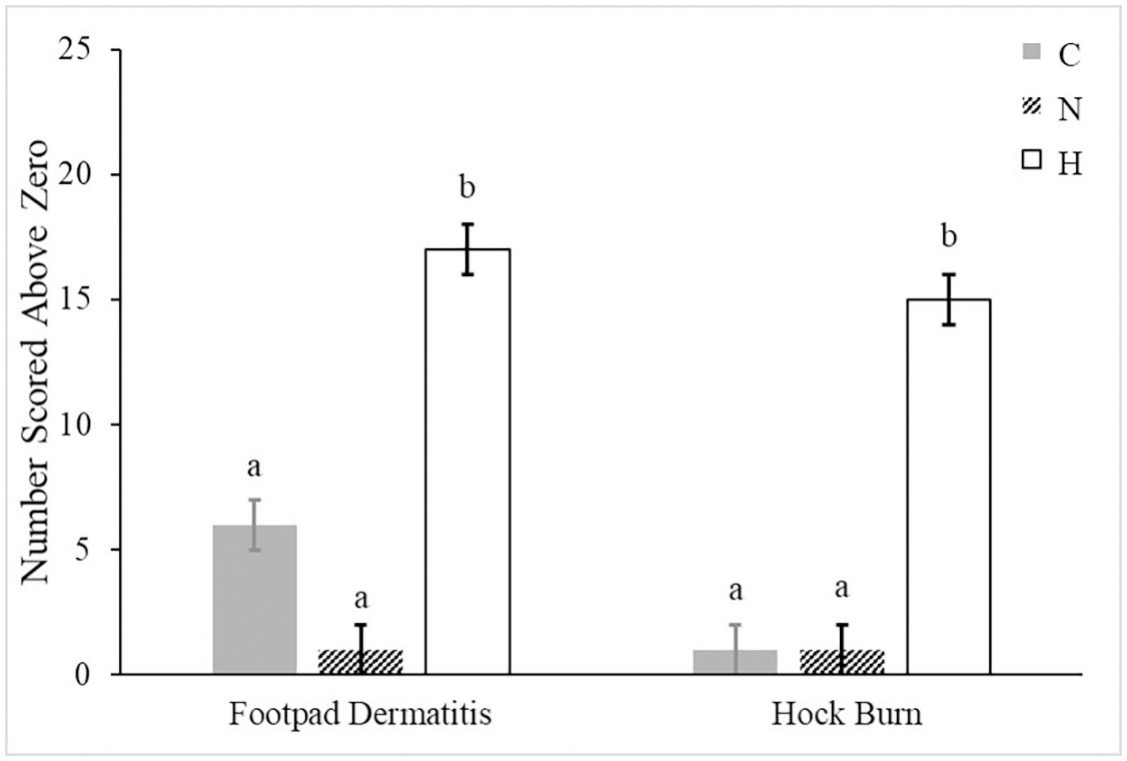
Number of birds (±SE) scored above zero for footpad dermatitis and hock burn on male broilers (10 birds/pen) from pens receiving no supplementation (control, C, n = 5), normal silicon supplementation (0.011 ml supplement/kg BW, normal, N, n = 5), or high silicon supplementation (0.063 ml supplement/kg BW, High, H, n = 5) using typical industry scoring systems taken at 42 d. ^ab^Bars lacking a common superscript differ (*P* < 0.05).

On d 42, groups scored similarly for toe damage and keel blisters. Footpad dermatitis and hock burn varied by treatment (*P* = 0.002 and *P* = 0.007, respectively; Fig 4). High birds scored higher in both measures than in N or C (*P* < 0.05 for both).

### Bone metabolism markers and serum minerals

Treatment did not affect OC (C: 203 ± 8 μμg/ml; N: 196 ± 9 μμg/ml; H: 192 ± 8 μμg/ml) nor PYD (C: 12 ± 1 nmol/l; N: 11 ± 1 nmol/l; H: 12 ± 1 nmol/l). Serum silicon did not differ among C (1.25 ± 0.02 μμg/ml), N (1.20 ± 0.02 μμg/ml), or H (1.21 ± 0.02 μμg/ml), but supplementation reduced serum boron (*P* < 0.001) and increased serum calcium (*P* < 0.001). For serum boron, N (0.24 ± 0.01 μμg/ml) was lower than H (0.28 ± 0.01 μμg/ml; *P* < 0.001), and C was higher than either supplemented group (0.32 ± 0.01 μμg/ml; *P* < 0.001 for both comparisons). For serum calcium, C and N were similar (98 ± 2 μμg/ml and 100 ± 2 μμg/ml, respectively), but both were lower than H (106 ± 2 μμg/ml; *P* < 0.01 for both comparisons).

### Bone measures

**Fig 5:**
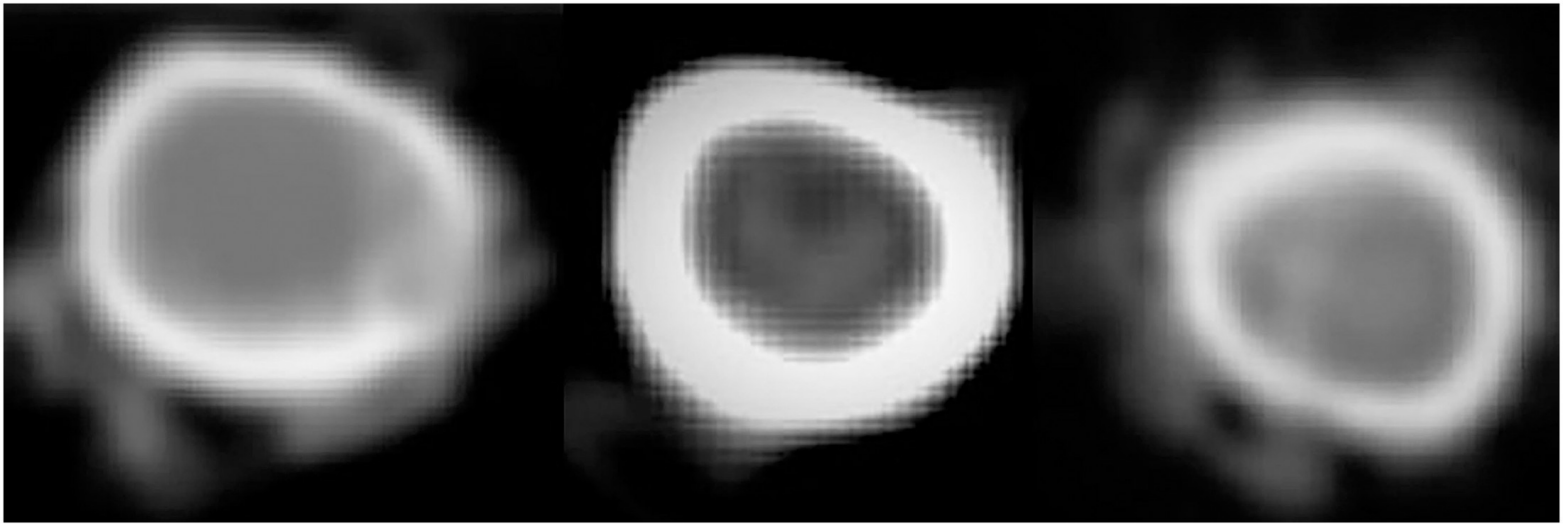
Cross-sections of right tibiae of male broilers from pens receiving either no supplementation (control, left), normal silicon supplementation (normal, middle), or high silicon supplementation (high, right) taken distally (Fracture End) from computed tomography scans. There were no differences in inner and outer diameters, cortical areas, and cross-sectional areas among groups at either fracture start or end. Scans were obtained just prior to four-point bending.

Neither total ash nor ash percentage in the femur and tibia were affected by treatment. Calcium concentrations in bone ash did not differ by treatment, but total calcium was lower in N (1,255 ± 43 mg) than in C (1,409 ± 34 mg, *P* = 0.02) and tended to be lower than in H (1,380 ± 34 mg, *P* = 0.07). Fracture start and end inner and outer diameters, cortical area, and cross sectional area were similar among treatments (Table 1; Fig 5). Fracture start slice density was greater in C and H than in N (*P* = 0.03, Table 2), but fracture end slice density was similar across groups. At fracture start, C had greater dorsal density than N (*P* = 0.04), but H was similar to both groups, and there were no differences among groups in any other cortex. Fracture end lateral cortical density tended to be highest in C than in N and H (*P* = 0.07), but treatment did not affect any other cortex at fracture end. Flexural rigidity tended to be greater in H (*P* = 0.07) when compared to C and tended to be similar to N (*P* = 0.09); no other breaking variable was different among groups (Table 3).

**Table 1:** Right tibiae morphological measures of male broilers (10 birds/pen) from pens receiving either no supplementation (control, n = 5), normal silicon supplementation (Normal, 0.011 ml supplement/kg BW, n = 5), or high silicon supplementation (high, 0.063 ml supplement/kg BW, n = 5) taken proximally (fracture start) and distally (fracture end) according to fracture location after breaking.

	Location of Measurement	Group		
		Control	Normal	High
**Bone Length (mm)**		110.3 ± 0.7	108.3 ± 0.7	109.9 ± 0.7
**Dorsopalmar Outer Diameter (mm)**	Fracture Start	9.6 ± 0.2	10.3 ± 0.2	9.8 ± 0.2
	Fracture End	9.0 ± 0.1	9.0 ± 0.1	8.9 ± 0.1
**Dorsopalmar Medullary Diameter (mm)**	Fracture Start	6.0 ± 0.2	6.1 ± 0.2	6.5 ± 0.2
	Fracture End	5.0 ± 0.1	5.0 ± 0.1	5.1 ± 0.1
**Lateromedial Outer Diameter (mm)**	Fracture Start	11.4 ± 0.2	11.8 ± 0.2	11.5 ± 0.2
	Fracture End	10.4 ± 0.1	10.6 ± 0.1	10.5 ± 0.1
**Lateromedial Medullary Diameter (mm)**	Fracture Start	7.3 ± 0.3	7.5 ± 0.3	7.4 ± 0.3
	Fracture End	6.1 ± 0.2	6.1 ± 0.2	6.3 ± 0.2
**Cortical Area (mm**^**2**^**)**	Fracture Start	52.7 ± 1.5	54.3 ± 1.4	56.1 ± 1.4
	Fracture End	51.0 ± 1.1	52.2 ± 1.1	50.5 ± 1.1
**Cross Sectional Area (mm**^**2**^**)**	Fracture Start	89.1 ± 4.0	98.6 ± 4.0	91.1 ± 4.0
	Fracture End	73.9 ± 1.8	74.7 ± 1.8	74.6 ± 1.8

Scans were obtained just prior to four-point bending.

**Table 2:** Right tibiae density measures (mg calcium hydroxyapatite/cm^3^) of male broilers (10 birds/pen) from pens receiving either no supplementation (control, n = 5), normal silicon supplementation (normal, 0.011 ml supplement/kg BW, n = 5), or high silicon supplementation (high, 0.063 ml supplement/kg BW, n = 5) taken proximally (fracture start) and distally (fracture end) according to fracture location after breaking.

	Location of Measurement	Group		
		Control	Normal	High
**Slice Density**	Fracture Start	549 ± 13^a^	503 ± 13^b^	540 ± 13^a^^b^
	Fracture End	617 ± 9	601 ± 9	609 ± 9
**Lateral Cortical Density**	Fracture Start	530 ± 16	499 ± 16	513 ± 16
	Fracture End	635 ± 14	601 ± 14	591 ± 14
**Medial Cortical Density**	Fracture Start	540 ± 15	494 ± 15	519 ± 15
	Fracture End	611 ± 14	600 ± 14	605 ± 14
**Dorsal Cortical Density**	Fracture Start	547 ± 15	494 ± 15	529 ± 15
	Fracture End	578 ± 15	553 ± 15	546 ± 15
**Palmar Cortical Density**	Fracture Start	528 ± 16	483 ± 16	510 ± 16
	Fracture End	603 ± 16	565 ± 15	574 ± 16

^ab^Values within a row lacking a common superscript differ (*P* < 0.05).

Scans were obtained just prior to four-point bending.

**Table 3:** Right tibiae biomechanical measures of male broilers (10 birds/pen) from pens receiving either no supplementation (control, n = 5), normal silicon supplementation (Normal, 0.011 ml supplement/kg BW, n = 5), or high silicon supplementation (high, 0.063 ml supplement/kg BW, n = 5) taken proximally (fracture start) and distally (fracture end) according to fracture location after breaking.

	Location of Measurement	Group		
		Control	Normal	High
**Fracture Force, N**		448 ± 8	462 ± 8	464 ± 8
**Young’s Modulus, x 10**^**7**^ **N/mm**^**2**^	Fracture Start	1.4 ± 0.1	1.4 ± 0.1	1.5 ± 0.1
	Fracture End	1.7 ± 0.1	1.8 ± 0.1	1.9 ± 0.1
**Moment of inertia, mm**^**4**^	Fracture Start	43.4 ± 2.5	45.3 ± 2.5	43.8 ± 2.5
	Fracture End	33.4 ± 1.5	34.8 ± 1.5	33.4 ± 1.5
**Flexural Rigidity, x 10**^**8**^ **N**·**mm**^**2**^	Fracture Start	5.6 ± 0.2	5.6 ± 0.2	6.3 ± 0.2
	Fracture End	5.6 ± 0.2	5.6 ± 0.2	6.3 ± 0.2

One Control tibia was excluded from analysis due to breaking during scanning.

Flexural rigidity tended to be greater in H (*P* = 0.07) when compared to C and tended to be similar to N (*P* = 0.09) in Tukey’s *post hoc* analysis; no other breaking variable was or tended to be different among groups.

### Texture and cooking losses

Overall, treatment did not affect firmness as C (2,200 ± 163 g force) was similar to N (2,223 ± 168 g Force) and H (2,327 ± 194 g Force). Toughness was also similar among C, N, and H (8,791 ± 752 g force/sec, 9,097 ± 795 g force/sec, and 9,862 ± 971 g force/sec, respectively). Cooking losses were similar among groups, and there were no differences for pre- or post-cooking weights.

## Discussion

Although silicon supplementation seemed promising from previous research, the current study did not show improved bone measures with bioavailable silicon supplementation. Texture remained unaffected by silicon supplementation as well. However, high silicon supplementation worsened welfare scores that dealt with skin irritation, potentially due to the increased water consumption.

Water consumption tended to be higher in supplemented groups as compared to controls, despite similar feed consumption among groups. The supplement provided in their water was stabilized using propylene glycol, which has a faintly sweet taste. In an early experiment, the addition of sucrose to water increased consumption [37], but a later experiment found that sucrose concentrations ≥ 1 M produced an aversion response and decreased water consumption [38]. It is likely that the supplement added a slightly sweet taste to the water, enticing the birds to drink more or more often than their unsupplemented counterparts.

Supplementing silicon at a high concentration appeared to influence the formation of footpad dermatitis and hock burn. In commercial operations, over 45% of birds may score a 1 or above [6] on the same scale used in the current study [33]. Since footpad dermatitis can affect weight gain, welfare, and paw quality [39], high bioavailable silicon supplementation appears to increase the incidence these lesions, contrary to previous studies. Silicon can stimulate type 1 collagen synthesis [40, 41], affecting collagen quality in the skin and other soft tissues [29, 42]. Calomme and Vanden Berghe [29] found higher hydroxyproline content in the dermis of calves supplemented orthosilicic acid, indicating a greater amount of collagen. Despite high supplementation, results from the current study may indicate a greater synthesis rate does not lead to greater collagen quality. Additionally, the tendency for supplementation to increase water consumption, even in H as compared to N, may have contributed to more litter moisture. Litter moisture can increase the incidence of footpad dermatitis [39]. Water consumption and wastage likely contributed to greater litter moisture which in turn could have influenced the development of footpad dermatitis in H.

While orthosilicic acid supplementation can increase osteoblast differentiation and osteocalcin concentration *in vitro* [41], the current study found no difference in osteocalcin concentrations in supplemented broilers as compared to controls, either due to the supplemental concentrations or differences in responses to silicon between sexes. High levels of silicon may also induce premature apoptosis in osteoblast-like cells *in vitro* [43], ultimately reducing activity and indicating a beneficial range that the current study exceeded. However, this effect shown in Shie et al. (2011) may have more to do with hyperosmolality of culture medium than direct effects of silicon. There is some evidence that silicon supplementation may be more beneficial for females than males. Most studies examining ovariectomized animals have found positive effects of silicon [22, 44], while studies using exclusively male subjects have reported no differences in bone with silicon supplementation [27, 45, 46]. In male rats, a strong negative correlation between serum osteocalcin and increasing doses of silicon can occur without similar effects in females despite similar osteocalcin concentrations [23]. Likely, this sex effect contributed to the similar concentrations among groups in the current study. Using only male broilers may have limited the results of the current study, but male broilers are more common in industry due to their larger size.

Although serum silicon was unchanged by treatment, supplementation lowered boron concentrations in both treated groups and raised Ca in H. While serum B concentrations were lower in supplemented groups, the supplement provided 784 μμg B/ml. In the current study, serum B concentrations were higher than values reported in humans (20–75 ng/ml) [47] and similar to values reported in humans receiving supplementation (68–659 ng/ml) [47], indicating that Si supplementation did not inadvertently cause B deficiency. In rats and calves, silicon intake has been associated with increased serum Ca in calves and mice [29, 48] but not in horses [31] or turkeys [45]. Higher concentrations of serum Ca may indicate greater availability for deposition into bone, but Ca concentrations within bone did not differ among groups in the current study. These results support previous findings on the effects of silicon supplementation in Ca and Mg metabolism as greater serum Ca concentrations in the treated group did not lead to higher tissue concentrations [48]. Though one study showed silicon supplementation and higher serum silicon affected femoral and vertebral mineral composition in male rats [49], other studies, including the current one, have not shown similar increases in bone minerals in males [23, 27, 45]. Since silicon supplementation can decrease Mg retention without affecting Ca metabolism [46] and reduce Al absorption [50], silicon may interfere with the absorption of other trace elements causing lower serum concentrations. However, these differences in serum concentrations may indicate increased tissue uptake [48] or may not translate to similarities in bone mineral composition or density as seen with this study and others [46, 49].

The exact mechanism of gastrointestinal silicon uptake is still unknown [24, 51], though silicon source matters for absorption and retention [31, 52]. Silicon sources may differ in their degradation and dissolution throughout the gastrointestinal tract [52, 53] as well as affect intestinal retention [54], potentially suggesting a route of paracellular absorption across the lumen as monomeric forms are most easily absorbed [52, 55, 56]. Additionally, orthosilicic acid may bind with other minerals, such as B, Al, or molybdenum, changing absorption and retention [56]. However, most studies have only examined changes in mineral balance, including Si, through intake and excretion values [31, 46, 52, 56] which, while informative, do not provide a mechanism for Si absorption or potential interaction with other minerals which may affect bone and other tissues.

Fracture force at breaking was similar across treatments, mirroring previous findings in turkeys and rats [45], as well as water silicon supplementation to broilers [27]. However, these findings contradicted other studies showing greater breaking strength in silicon-supplemented animals [26, 28, 57]. This disparity was likely due to differences between four-point and three-point bending in bone mechanical testing [58] as three-point bending generates high shear stress and four-point bending ensures almost no shear stress where loading is applied [36]. Because four-point bending generates no shear stress, it is preferential for measuring mechanical properties.

In all the studies supplementing silicon to broilers, the amounts used in the current study, 0.35 mg silicon/kg BW for N and 1.98 mg silicon/kg BW for H, most closely match those in the study by Sgavioli et al. [27]. The previous study found differences in phosphorus, zinc, and manganese concentrations of tibiae above non-supplemented birds beginning at 0.35 mg silicon/kg BW but no differences in strength or density even at the highest level of supplementation, 0.53 mg silicon/kg BW. Other studies used much higher rates of supplementation. In the study by Scholey et al. [26], bone strength was improved in birds supplemented approximately 45 mg silicon/kg BW, and tibia ash was increased in birds receiving a minimum 7.7 mg silicon/kg BW. Nakhon et al. [28] showed increased tibia breaking strength in birds supplemented 12.7 mg silicon/kg BW but not in birds supplemented 17.8 mg silicon/kg BW. Intakes of 47 to 106 mg/kg BW/d produced greater tibial BMD and tended to increase serum OC in female rats [23]. Each of these studies used different rates and methods of supplementation and reported silicon either as percent of supplement or mg/kg included in the diet, making standardization difficult.

Current estimated dietary intakes in humans range from 20–50 mg Si/d [24] with the recommended intake being 10–25 mg Si/d [59], meaning that an adult’s intake on a kg BW basis [60] may vary from 0.3 to 0.8 mg Si/d. This range is below the highest level of supplementation in the current study and well below levels demonstrating positive influences on bone through changes in BMD or strength. To reach even the minimum concentration of silicon which showed an increase in tibia ash [26], adults would need to consume between 444 and 621 mg Si/d. The differences between fed amounts in experiments and recommended intakes in humans demonstrate the need for better standardization in the literature and potentially a reevaluation of current recommendations. Standardizing Si supplementation and intakes on a kg BW basis would make translating research across species easier and improve interpretation of results. While current recommendations may be based on the essentiality of Si for growth [59, 61, 62], recommendations for positive influences on bone may need to be substantially higher than the current 10–25 mg Si/d.

## Conclusions

Though deficiency will negatively affect bone, the results of this study suggest that the silicon supplementation in the form and at the rate provided does not improve bone quality in male broilers. Even when supplemented at high concentrations in a bioavailable form, silicon did not improve bone density, morphology, or breaking strength above controls. The highest level of supplementation in the current study was below concentrations used in studies demonstrating silicon’s positive influence on bone, indicating that previously suggested minimum thresholds for intake need to be reevaluated. Regardless, factors such as growth rate and mechanical loading likely play a greater role in developing bone quality than trying to supplement on top of good basic nutrition alone.
